# Examining Preference Heterogeneity in Best-Worst Scaling: Case of Preferences for Job Opportunities in Artisanal Small-Scale Gold Mining (ASGM) Communities in Indonesia

**DOI:** 10.3390/ijerph19010306

**Published:** 2021-12-28

**Authors:** Satoru Komatsu, Yayu Isyana D. Pongoliu, Masayuki Sakakibara, Taro Ohdoko

**Affiliations:** 1 Graduate School of Global Humanities and Social Sciences, Nagasaki University, Nagasaki 852-8521, Japan; 2 Faculty of Economics, State University of Gorontalo, Gorontalo 96128, Indonesia; yayuidp@gmail.com; 3 Research Institute for Humanity and Nature, Kyoto 603-8047, Japan; sakaki@chikyu.ac.jp; 4 Graduate School of Science and Engineering, Ehime University, Matsuyama 790-8577, Japan; 5 Faculty of Economics, Dokkyo University, Saitama 340-0042, Japan; ohdoko@dokkyo.ac.jp

**Keywords:** ASGM, best-worst scaling, job opportunities, preferences, Indonesia

## Abstract

This research empirically examines the preferences for job-related attributes among rural villagers living close to artisanal and small-scale gold mining (ASGM) in Indonesia. Based on hypothetical scenarios in which a private company collaborates with the local government to establish a food processing industry in these villages, a questionnaire survey designed with best–worst scaling (BWS) was administered to households to determine their preferences. Additionally, the heterogeneity among the villagers was examined by applying a latent class logit (LCL) model. The main household survey was conducted in 2019 in Bone Bolango Regency, Gorontalo Province. The estimation results revealed that villagers are separated into four classes, and each class has different and unique preferences. Creating more job opportunities for society is a highly evaluated attribute; however, the preference for skill acquisition differs among groups. The results indicate that accounting for heterogeneous preferences regarding job opportunities is helpful to delink dependency on ASGM and health hazards and improve the livelihoods of rural villagers. The study yields key information to substantially reduce environmental and health hazards in the poverty-plagued ASGM community by facilitating job opportunities in Indonesia.

## 1. Introduction

The release of mercury and its components into the atmosphere has had serious impacts on human health and the local environment. One of the major activities that emits mercury (Hg) is artisanal and small-scale gold mining (ASGM), an activity in which individuals or small groups extract gold using simple tools and methods. Commonly, mercury is used to extract gold from gold ores to make an amalgam, which is then burned to distill mercury and isolate gold. Mercury is released to the air, water and soil during the vaporization and tailing process and is diffused into the environment.

ASGM is the predominant source of anthropogenic mercury emissions, accounting for 37.7% of the total mercury emissions in 2015 [1]. ASGM plays a major role in gold extraction; it is estimated to constitute 17–20% of global gold production [2]. The release of Hg from ASGM is higher in countries with lower levels of technology [3].

ASGM practices have expanded, especially in many low- and middle-income countries, and have caused occupational mercury intoxication in local societies. Approximately 15 million people, including approximately 3 million women and children, engage in ASGM activities in developing countries [4]. Seccatore et al. estimated that over 16 million miners were involved in gold mining worldwide [3]. Gold extraction in ASGM is commonly informal, and ASGM causes widespread mercury contamination for miners and surrounding villagers. Globally, 3.3 to 6.5 million miners suffer from moderate levels of chronic metallic mercury vapor intoxication [5]. Given the impacts of ASGM on the rural economy and the mercury consumption in this sector, the sustainable management of the ASGM sector is crucially important to achieve sustainable economic growth while preventing environmental degradation and reducing health risk.

Engagement in this environmentally and occupationally hazardous activity is closely associated with chronic poverty in rural societies, where income-generating opportunities are limited. In places where job opportunities are limited and unstable, obtaining cash through extracting gold is crucial for sustaining day-to-day livelihood. Even if villagers are not directly engaged in mining, mining operations have effects on other industries, such as shops, restaurants, driving and agriculture. Therefore, the local economy where ASGM is located is connected to the mining sector.

There are two ways to reduce poverty and to improve local livelihoods in existing ASGM communities. The first way is to minimize the extensive environmental and health hazards of ASGM. Various previous studies have attempted to replace mercury amalgamation or cyanide processing, which also poses safety and health concerns (refer to [6], for example). Alternative technologies can address safety and health concerns, but they require users to have prior knowledge and technical training [6]. Major technological transformations usually require continuous effort to develop the capacity of miners in the long run. Alternative technologies should be affordable, cost-effective, and available when necessary. Moreover, technological development cannot directly overcome the underlying reality of rural villages, i.e., pervasive poverty.

Another way is an alternative livelihood approach. Alternative livelihood approaches in the context of mining assume that farmers can cease or reduce their reliance on ASGM if livelihood-based policy interventions, such as the development of plantations, are effective in generating sufficient cash income. If such interventions are successful, alternative job opportunities that generate cash income are promising to eradicate chronic poverty and to reduce dependency on ASGM.

Alternative livelihood approaches have extensively been applied in the context of biodiversity conservation to convert conventional livelihoods that have substantial impacts on biodiversity into sustainable livelihoods that reduce resource consumption. Examples of alternative livelihoods are ecotourism in Nepal [7] and the provision of microcredit for villager groups in Indonesia [8]. Based on an extensive review on the effects of alternative livelihood initiatives on biodiversity conservation, Roe et al. found mixed outcomes; nine initiatives reported positive conservation outcomes, another nine reported no change, and the remaining three reported negative outcomes [9].

However, to date, various studies have pointed out that alternative livelihood approaches in the field of mining fail to incentivize the abandonment of mineral extraction. Empirical findings on the expansion of agriculture as a livelihood intervention tool in Myanmar concluded that mining and agriculture are unlikely to be substitute livelihood options for villagers; rather, they are complementary options [10]. Agriculture and mining were found to be complementary in Sierra Leone, where inhabitants engage in farming in rainy seasons and mining in dry seasons [11].

How can we achieve the success of alternative livelihood approaches to attain the multifaceted goals of eliminating rural poverty and minimizing environmental hazards? Answering such questions is difficult; however, we assert that bottom-up approaches that incorporate the latent preferences of miners and villagers are crucially important. Alternative livelihoods are presumed to be a promising opportunity that can absorb local labor forces and yield sufficient income, in addition to meeting the latent demands of potential local workers. Collecting and incorporating possible job seekers’ attitudes and opinions on alternative job opportunities are the basis for formulating effective policies. Alternative livelihoods are effective if a series of income-earning activities can prevent people from pursuing employment in ASGM and are attractive for individuals already engaged in mining activities [12]. Therefore, for proposing attractive alternative livelihoods and addressing local public health issues, it is critical to clarify the latent demand for job opportunities of rural villagers who may otherwise work at ASGM sites or surrounding industries.

To date, a limited number of case studies have evaluated preferences for job opportunities in the context of ASGM. A conference presentation appears to be the first attempt to analyze citizens’ preferences for possible job opportunities in Indonesia [13]. The study conducted household surveys to elicit job preferences through best–worst scaling (BWS) and applied a mixed logit model to examine preferences for job opportunities [13].

This paper applies the data set used in [13] and extends it to examine the source of preference heterogeneity from various socioeconomic variables (household income, dependency on mining, education, residency, etc.). If the source of heterogeneity is successfully and rigorously identified, this information can support policymakers in designing alternative livelihood policies.

BWS is frequently applied to examine the relative importance of attributes in the field of health economics and marketing and is increasingly applied in environmental and resource economics. BWS was first introduced by Louviere and Woodworth in a working paper in 1990, while Finn and Louviere published the first application of the technique [14]. Commonly, respondents are asked to select their most preferred choice and their least preferred choice in a choice set. Extensive literature has examined the relative importance of policy measures and opinions on environmental attributes as well as latent attributes among stakeholders.

This research empirically examines the preferences for job-related attributes among rural villagers living close to ASGM in Gorontalo Province of Indonesia. Based on hypothetical scenarios in which a private company collaborates with the local government to establish a food processing industry in the villages, the research applies BWS techniques to determine potential workers’ latent demands for job opportunities. Given the heterogeneous livelihoods of the villagers, the study also examines preference heterogeneity by applying a latent class logit (LCL) model. This research aims to expand the discussion and provide new insights into job opportunities to reduce dependency on ASGM and improve public health in poverty-plagued villages in Indonesia.

## 2. ASGM Operations in Indonesia

Indonesia emits the second largest amount of mercury in the world [15]. ASGM operations have spread widely in various provinces in Indonesia. There are limited reliable sources of data, but the estimated number of workers in artisanal and small-scale mining in Indonesia is 109,000 [16]. Note that this number includes workers engaged in extracting a broad range of minerals, including gold. Ten percent of workers involved in artisanal and small mining operations are women and children [16]. Extensive previous studies have described the economic benefits accrued from mining operations and noted the mercury contamination in ASGM and surrounding areas as well as safety and health hazards. Research comparing development benefits of ASGM and large-scale mining in North Sulawesi found that ASGM brought substantial benefits for the local community in the form of a decreased unemployment rate, increased income, better infrastructure, and increased market diversification [17]. At the same time, a high level of mercury contamination has been reported in ASGM communities in various provinces, such as in West Java [18], West Nusa Tenggara [19], Buru Island, and Mollucas [20].

Gorontalo Province, located on northern Sulawesi Island, is one of the typical provinces where ASGM activities have deeply penetrated the economy. Similar to the literature examining other provinces, studies conducted in Gorontalo have pointed out adverse effects of ASGM on the environment and human health. Mining operations in Hulawa villages in Gorontalo Utara Regency date back to the 19th century, whereas mining operations in Ilangata villages began just 15 years ago [15]. Inhabitants living close to ASGM sites experience higher hair mercury contamination than those living in non-ASGM sites [21]. Based on the results of spirometry tests, miners suffer from lung disorders after exposure to evaporated mercury [22]. Part of the context of these ASGM activities is the limited number of prospective industries that can absorb labor in Gorontalo, hindering sustainable development, particularly in rural villages.

Bone Bolango Regency, located in the Gorontalo Province in Indonesia, is a location where many ASGM extraction sites are located. The livelihoods of the inhabitants of the Bone Bolango Regency depend on the Bone River. The inhabitants use the river’s water as a source of drinking water and for cooking and agricultural production. Fisheries are common along the Bone River; inhabitants commonly consume fish and shrimp caught from the river. A recent survey noted heavy metal contamination in the Bone River. Concentrations of As (arsenic), Hg, and Pb (lead) in water samples were found to exceed the water safety standards defined by the World Health Organization [23]. Contamination by heavy metals is due to ASGM activities [23].

In rural Bone Bolango, opportunities to obtain cash income are limited to agriculture, fisheries, chauffeuring, and retail work, among other activities. In particular, households with low educational attainment face limited options for obtaining cash income; they are more dependent on mining, probably due to the limited job opportunities in the formal sector in Gorontalo [24]. Generating alternative livelihoods that can incentivize rural villagers to avoid mining activities is key to achieving multifaceted development goals, namely, avoiding environmental pollution, mitigating adverse health effects, and improving rural livelihoods.

This research assumes that the food processing industry in Gorontalo is promising to absorb the labor force and effectively utilize local agricultural production. In Gorontalo, agriculture is the second largest economic sector [25]. Currently, the economy of Gorontalo relies mainly on agriculture, which produces primary commodities (rice, maize). Corn is a commodity that is commonly planted and dominates dryland agricultural activity in Gorontalo [25]. Research notes that the development of the food processing industry can facilitate more production and increase the value added of cultivated commodities. Gorontalo has the potential to develop small and medium enterprises for maize products given the availability and affordability of the labor force and raw materials [26]. The development and use of technologies that can be adopted for the corn processing industry can benefit the economic profile of Gorontalo [27]. Considering the economic profile and prospects of the food processing industry, it is important to examine the potential of this industry from the perspective of the potential labor supply.

The objectives of this research are to examine job-related attributes that are highly evaluated by rural villagers in Gorontalo, Indonesia. Based on hypothetical scenarios that present opportunities to work in the food processing industry, the research examines the preferences of villagers in relation to the following seven attributes: contribution to local environmental quality, frequency of payments, employment of friends in the same company, occupation-related health risk, creating more job opportunities for society, reputation of the company, and acquisition of new skills. Estimation results obtained from the collected information are expected to support the encouragement of alternative livelihoods and minimize dependency on mining.

## 3. Methodology

### 3.1. Survey Areas and Questionnaire Design

The survey was conducted in Bulawa and Suwawa Timur districts in the Bone Bolango Regency in Gorontalo Province in September 2019 (refer to [13] for the specific names of the surveyed villages of Bulawa and Suwawa Timur districts). The households were visited based on the map created from satellite imagery to cover households in the surveyed districts and to minimize sampling biases. In the field survey, not every identified household could be the location of a survey because these buildings are not always utilized for residential purposes and sometimes they house governments or serve other purposes. The research collected data from newly identified residential households via field observations that were not identified from satellite imagery. During the survey, field investigators identified residential properties whose household members were not at home. Investigators also identified households whose adult members were not at home. Since the household survey was conducted beginning in the early morning, if respondents were not at home, the households were revisited at night. We did not collect data on rejection rate; however, most of the household members were very responsive and kind to the investigators if they understood the objectives of the survey; thus, rejection was rare. In this sense, sampling biases due to rejection were limited.

The survey was administered to 503 villagers, and a sample of 91 villagers who were the household heads served as the sample for this paper. Most of the answers were collected from the wife of the household, who was unlikely to be a job seeker. To examine the consistency of the results, the authors checked that similar estimation results could be obtained when answers from non-household heads were incorporated.

Prior to administering the survey, the investigators received training to ensure that they understood the survey objectives and questionnaire design. Since the literacy rate of rural households was considered low due to lower educational attainment, answers from respondents were collected through individual face-to-face interviewer-administered elicitations. To obtain reliable and representative answers from households, the investigators sought respondents who were the head of their household or served as the household’s decision maker.

Table 1 shows the attributes selected to elicit job preferences. To examine the validity of each attribute, this research refers to a previous survey on job preferences conducted in 2017 in rural areas of Gorontalo. Accounting for the local characteristics in the ASGM region, environmental and health attributes were incorporated into the survey. Income-based attributes on job preferences, such as a periodic increase in salary and higher income than that offered by other companies, are generally evaluated higher than other attributes. Income-based attributes were not included in the questionnaire to optimize identification of additional relevant attributes affecting job preferences.

Respondents were presented with the following hypothetical scenarios.

*Suppose that a private company in collaboration with the local government establishes a food processing industry near the market of Kecamatan. The company is going to hire local people for food processing duty. Monthly payments are equivalent to those of the other companies in the same industry in Gorontalo Province, but you are expected to receive more compensation as you gain experience. Suppose that you receive a job opportunity in that company. If you decide to work there, you must work there as a full-time employee, meaning that you cannot work full-time for other enterprises. Which factors (attributes) do you think are the most important and least important in deciding to accept a new job opportunity?*

**Figure 1:**
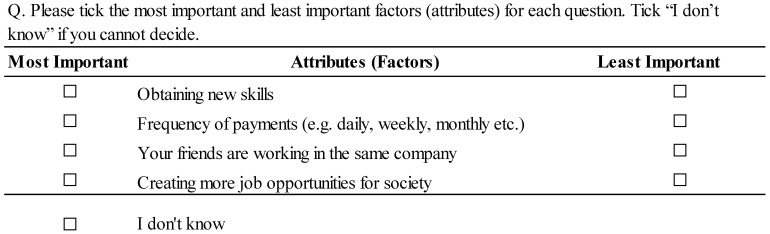
Sample questions.

One of the sample choice sets is shown in Figure 1. Each respondent was asked to choose what he or she considered the best and worst of the four attributes. Each respondent repeated this exercise seven times with different choice sets. Respondents were instructed to select “I don’t know” if they could not understand the question or decide on their answers.

To construct BWS questions, this research employed a balanced incomplete block design (BIBD) to ensure that each alternative appeared an equal number of times and was equally paired with each of the other alternatives across all choice sets (refer to Street and Burgess [28] and Hinkelmann and Kempthorne [29]).

### 3.2. Estimation Procedures

To examine latent preferences obtained from BWS, maximum difference (maxdiff) models are commonly applied, and simple counting methods are used to obtain the best-minus-worst score [30,31]. The basic theory of the maxdiff model is random utility maximization [32,33]. Suppose a random utility of choosing i as the best item and $i^{\prime }$ as the worst $U_{ii^{\prime }}=\left[V\left(i\right)-V\left(i^{\prime }\right)\right]+\varepsilon_{ii^{\prime }}/\varphi$, where V(·) denotes the deterministic component of indirect utility, $\varepsilon_{ii^{\prime }}$ is the *iid* (independently and identically distributed) error component, and φ is the scale parameter, which is inversely proportional to the standard deviation of the error component. When the error component $\varepsilon_{ii^{\prime }}$ depends on an independent standard Gumbel or type I extreme value distribution, an individual choice probability can be formed from the well-known form of a multinomial or conditional logit model, as shown in Equation (1), representing the maxdiff model:

(1) $$P\left(ii^{\prime },i\neq i^{\prime }|M\right)=\frac{exp\left(\varphi \left(V\left(i\right)-V\left(i^{\prime }\right)\right)\right)}{\sum_{\begin{array}{c} j,j^{\prime }\in M\\ j\neq j^{\prime } \end{array}}^{}exp\left(\varphi \left(V\left(j\right)-V\left(j^{\prime }\right)\right)\right)}$$

where *M* denotes the options, which consist of the choice set provided to the respondents.

To examine data collected from BWS that contain “best” and “worst” answers, a random utility function that assumes a linear-in-parameter form, as shown in Equations (2) and (3), has been frequently used:

(2) $$U\left(ii^{\prime },i\neq i^{\prime }\right)=\alpha_{i}x_{i}-\alpha_{i^{\prime }}x_{i^{\prime }}+\varepsilon_{ii^{\prime }}/\varphi$$

(3) $$U^{*}\left(ii^{\prime },i\neq i^{\prime }\right)=\varphi U\left(ii^{\prime },i\neq i^{\prime }\right)=\beta_{i}x_{i}-\beta_{i^{\prime }}x_{i^{\prime }}+\varepsilon_{ii^{\prime }}{}^{*}=\beta_{i}x_{i}+\beta_{i^{\prime }}\left(-x_{i^{\prime }}\right)+\varepsilon_{ii^{\prime }}{}^{*}$$

where $x_{i}$ and $x_{i^{\prime }}\;$ denote dummy variables that take a value of 1 when the item is chosen by the respondent and 0 otherwise. $\alpha_{i}$ and $\alpha_{i^{\prime }}$ are the true marginal utility parameters. $\beta_{i}$ and $\beta_{i^{\prime }}$ are the parameters where the true marginal utility parameters, $\alpha_{i}$ and $\alpha_{i^{\prime }}$, and the scale parameter, φ, are jointly estimated; these 7 arameters are difficult to be estimated separately. In coding the data, we set the most important item as 1, the least important as −1, and all other items as 0.

When utilizing a multinomial logit model Equation (1), there are several strict assumptions to relax: preference homogeneity, a property of independence of irrelevant alternatives (IIA), and an uncorrelation of marginal utility parameters. We employ an LCL model to relax these model assumptions because of the anticipated heterogeneity in surveyed preferences [34,35,36]. Since any form of correlation can be permitted under LCL [37], we can control parameter correlations caused by behavioral phenomena and scale heterogeneity when we employ LCL. Refer to Appendix A for further discussion.

For the LCL model, the number of classes is specified exogenously. This research incorporates a number of variables in the membership functions, including dependency on mining, household income (natural log), age, education, residency, and duration of residency in the current location. Several information criteria have been utilized in previous papers. Boxall and Adamowicz [34] utilized the minimum Akaike information criterion (AIC) and the Bayesian information criterion (BIC). Hynes et al. [38] proposed employing AIC3 and corrected AIC (crAIC). We decided to utilize these four criteria to determine the number of classes with every combination of covariates to be considered.

## 4. Estimation Results

Table 2 shows the socioeconomic variables of the households. The average number of household members is 4.3. Annual income is IDR 36.542 million (USD 2583) per household, or IDR 10.65 million (USD 740) per capita, indicating that villagers are not wealthy. The share of agricultural income and mining income is approximately 20%, and other income sources include working in a shop, working as a driver, or engaging in freelance activities.

The estimation results of the conditional logit model are shown in Table 3. Reputation (reputation of the company) is set as a reference attribute; therefore, respective parameters should be interpreted as the relative importance compared with the reputation attribute. Estimation results indicate that job (creation of more job opportunities for society) is evaluated highest, and the friend attribute, i.e., employment of friends in the same company, is evaluated as least important. Based on the estimation results of the CL (Conditional Logit) model, villagers seek more job opportunities for society.

Table 4 shows the estimation results of the LCL model that assumes preference heterogeneity among different classes. Based on the information criterion, four class models with a constant membership function model were identified. The estimation results that included socioeconomic variables in the membership functions provided lower explanatory power than the estimation model presented in Table 4. The class share is 20.0% for class 1, 16.0% for class 2, 27.5% for class 3, and 36.5% for class 4.

Compared with the estimation results of the LCL model, Table 4 explains distinct features in the estimation results. Class 1 prioritized occupation-related health risks, followed by job opportunities, better local environmental quality, and skill acquisition. The employment of friends in the company and the frequency of payments were evaluated as less important. In contrast to class 1, class 2 evaluated frequency of payments highest, followed by health consciousness, job opportunities and better local environmental quality. Among the four classes, class 2 is the only class that did not positively evaluate skill acquisition. The employment of friends in the company is also evaluated as less important by this class.

Class 3 is quite unique. Skill acquisition is the only attribute that is positively evaluated by this class. Other attributes are not statistically significant with respect to the baseline attribute (reputation of the company).

Class 4 evaluated job opportunity highest, followed by better local environmental quality, frequency of payments, skill acquisition and health consciousness. The results are similar to those from the conditional logit model presented in Table 3. Since this class was the largest and the majority of respondents were in this category, this class reflected the average characteristics and its constant was applied as the reference for the membership function of the other three classes.

## 5. Discussion

The estimation results in Table 3 show that respondents attribute high importance to more local job prospects, flexibility in payment schemes, the company’s environmental consciousness, the company’s consciousness of occupation-related health risks, and opportunities to obtain skills. The estimation results of the LCL model indicate the need to account for preference heterogeneity. In Table 4, the most prevalent class is class 4, which comprises 36.5% of respondents, and the estimation results are similar to those in Table 3. However, the proportion of the respondents (27.5%) who are more likely to be categorized into class 3, which places importance on only skill acquisition, is not negligible, according to critical mass theory in sociology [42,43,44]. Therefore, even though job opportunities are highly evaluated by classes other than class 3, the unique features of class 3 need to be taken into account in managing ASGM issues in Gorontalo, Indonesia.

An attribute that is relatively highly evaluated overall is the creation of more job opportunities for society. This implies that limited job opportunities are a serious concern and an urgent issue for rural villagers in Bone Bolango. Although no classes evaluate environmental consciousness as the most important attribute, class 4 (the largest class) ranks it the second most important attribute. Therefore, heavy metal contamination is recognized by a non-negligible number of individuals as a serious local environmental issue that Bone Bolango faces.

Although class 2 is estimated to represent only 16.0% of the total, payment frequency received a great deal of attention from this class, which implies that these villagers want prospective companies to provide flexible salary payment options.

Contrasting features are found in the skill acquisition attribute. The estimation results indicate that skill acquisition at work received relatively low interest from villagers categorized in classes 1, 2, and 4. This may indicate that rural villagers have little experience receiving incremental salaries based on their acquired skills since jobs that require professional skills are scarce there. In contrast, class 3 evaluates only skill acquisition highly, meaning that members of this category evaluate whether opportunities to obtain skills are available. These data suggest that to some, the opportunity to acquire skills is crucially important to boosting income and improving living standards above all other preferences. It is important to examine this distinction in order to raise awareness of the importance of skill development for poverty eradication in rural Gorontalo.

The results for occupation-related health risk in Table 4 indicate unique characteristics. Villagers categorized into class 1 (20.0%) and class 2 (16.0%) prefer companies with consciousness of occupation-related health risks; however, respondents in class 4 (36.5%) place less importance on this attribute. There are no statistically significant differences for class 3 (27.5%), meaning that occupation-related health risks are not important compared with the baseline attribute (reputation of the company). If alternative livelihood approaches are implemented to alleviate health risks, such practices may not be widely accepted by potential employees.

The results obtained from the LCL model yield important policy implications for managing ASGM and improving public health in the survey regions because they revealed preference heterogeneity. This indicates that a single policy does not apply to all; several packages that assume heterogeneous preferences should be provided to meet the latent demands of villagers.

The research notes two limitations of generalizing results for establishing policies in ASGM communities to reduce environmental degradation. The first limitation is the generalizability of the findings. Our sampling procedures attempted to minimize sampling biases but are not considered random sampling because complete sample frames could not be prepared before the survey. The research cautions that existing sampling errors may influence the generalizability of the results. Second, the sampling could not focus on potential job seekers due to the lack of formal job markets. If sampling was conducted for only job seekers, the estimation results would yield better policy implication.

Since the research was conducted to examine latent preferences for job-related attributes, the level of attributes was not examined. For example, respondents would provide different opinions and preferences regarding the level of occupation-related health risks and possible permanent damages. It is necessary to conduct a stated preference survey to identify preferences on the level of those attributes, which can help formulate more detailed policy to minimize dependency on ASGM.

This research includes various sociodemographic variables in the LCL model membership function to find determinants segregating different classes of workers. If current miners are identified in alignment with specific classes, this allows for interpretation of heterogeneity of preferences for job opportunities and the consequent dependency on mining. Other observable socioeconomic variables, such as the duration of residency in the current location, affluence of households, and educational variables, cannot be included in the membership functions from the viewpoint of the information criterion; therefore, those indicators are not suitable for separating classes. This may indicate that unobserved variables, such as psychological variables, environmental concerns, and attitudes toward political parties, may be candidates for further research. Further examinations are required to verify other potential determinants.

Since this study is limited to case studies in Gorontalo Province, Indonesia, further research is pivotal for generalizing the results. As a case study, the present study is beneficial for policymakers considering the provision of job opportunities in rural areas to reduce dependency on mining and simultaneously improve rural livelihoods.

## 6. Conclusions

This research empirically examines the preferences for job-related attributes among rural villagers living close to ASGM in the Gorontalo Province of Indonesia. Based on hypothetical scenarios in which a private company in collaboration with the local government establishes a food processing industry in villages, the research applied BWS techniques to determine possible workers’ latent demands for job opportunities. It also examined the heterogeneity of the villagers by applying an LCL model. The estimation results indicated that villagers are separated into four classes, and each class has different and unique preferences. The results suggest different policy interventions to effectively capture the heterogeneous livelihoods of rural villagers. Accounting for heterogeneous preferences for job opportunities is helpful to delink dependency on ASGM and health hazards and improve the livelihoods of rural villagers.

## Figures and Tables

**Table 1:** Selected Preference Attributes.

Attributes	Description
Environment	Contribution to better local environmental quality
Frequency	Frequency of payments (e.g., daily, weekly, monthly)
Friend	Your friends are working in the same company
Health	Occupation-related health risk
Job	Creating more job opportunities for society
Reputation	Reputation of the company
Skill	Obtaining new skills

**Table 2:** Socioeconomic Variables of the Households (data collected from the household head only).

Variable		Sample Sizes	Mean	Std. Dev.	Min.	Max.
Number of household members (num.)		91	4.308	1.872	1	9
Household income	Total (per year, in million Rupiah)	91	36.542	45.147	0	270.06
	Per capita (per year, in million Rupiah)	91	10.465	16.079	0	115
	Share of agricultural income	88	0.195	0.340	0	1
	Share of mining income	88	0.172	0.322	0	1
Mining	Whether household has miner (1 = yes, 0 = no)	91	0.286	0.454	0	1
	Whether household head is miner (1 = yes, 0 = no)	91	0.220	0.416	0	1
	Number of miners per household (num.)	91	0.341	0.619	0	3
	Age (years)	88	46.466	12.641	21	79
	Years of education (years)	89	7.921	3.076	3	17
Head’s demographics	Status of residency in the current place of residence (1 = living in the household except occasional trip, 0 = living outside from household)	87	0.885	0.321	0	1
	Duration of stay in the current residential place (1 = 10 years or more, 0 = less than 10 years)	89	0.809	0.395	0	1

Note: 1 USD is equivalent to 14,148 Rupiah in 2019 [39]. Refer to [40,41] about descriptive statistics for codes (0, 1).

**Table 3:** Estimation Results of CL (Conditional Logit) model.

Factors	Coef.
Job	1.388 ***
	(0.114)
Frequency	1.046 ***
	(0.111)
Environment	1.008 ***
	(0.111)
Health	0.836 ***
	(0.110)
Skill	0.756 ***
	(0.109)
Friend	0.094
	(0.107)
Log-likelihood	−1351.604
Number of Observations	7164

Note: Standard errors are in parentheses. The symbol *** denotes statistical significance at the 1% levels.

**Table 4:** Estimation Results of LCL model.

		Class 1	Class 2	Class 3	Class 4
**Attributes**					
	Environment	1.462 ***	1.856 ***	−0.035	2.136 ***
		(0.399)	(0.428)	(0.222)	(0.278)
	Frequency	0.620	4.461 ***	0.005	1.950 ***
		(0.438)	(0.744)	(0.229)	(0.291)
	Friend	−0.160	−0.283	0.243	0.247
		(0.324)	(0.361)	(0.211)	(0.218)
	Health	2.682 ***	3.926 ***	−0.296	0.620 **
		(0.551)	(0.693)	(0.261)	(0.261)
	Job	1.809 ***	2.774 ***	0.028	3.302 ***
		(0.460)	(0.636)	(0.237)	(0.348)
	Skill	1.299 ***	0.444	0.732 ***	1.038 ***
		(0.371)	(0.345)	(0.237)	(0.250)
**Membership Function (reference = class 4)**					
constant		−0.602	−0.828	−0.283	
		(0.408)	(0.371)	(0.325)	
**Membership Probability**					
		0.200	0.160	0.275	0.365
	Log-likelihood	−1225.222			
	Number of Observations	7164			

Note: Standard errors are in parentheses. The symbols *** and ** denote statistical significance at the 1%, and 5 % levels, respectively.

## Data Availability

Since the household survey was conducted in mining areas, where security concerns and privacy restrictions exist, data are available on request.

## Appendix A

The choice probability of LCL is represented as follows:

$$P_{n}\left(ii^{\prime },i\neq i^{\prime }\right)=\sum_{s=1}^{S}\pi_{ns}\prod_{t=1}^{T}\frac{\exp\left(\varphi \left(V\left(nit|s\right)-V\left(ni^{\prime }\;t|s\right)\right)\right)}{\sum_{\begin{array}{c} j,j^{\prime }\in M\\ j\neq j^{\prime } \end{array}}^{}\exp\left(\varphi \left(V\left(njt|s\right)-V\left(nj^{\prime }\;t|s\right)\right)\right)}$$

$$\pi_{ns}=\frac{exp\left(c_{s}+\gamma_{s}{}^{\prime }\;z_{n}\right)}{\sum_{s^{*}=1}^{S}exp\left(c_{s^{*}}+\gamma_{s^{*}}{}^{\prime }\;z_{n}\right)}$$

where t denotes the choice occasion (maximum 7), s the class into which respondents are classified, $c_{s}$ is a class-s-specific constant, and $z_{n}$ is a vector of covariates with parameter vector $\gamma_{s}$. $c_{s}+\gamma_{s}{}^{\prime }z_{n}$ is a membership function, and $\pi_{ns}$ is the probability of membership. The Sth parameter vector is normalized to zero to secure identification. With the LCL model, we can estimate the shares of classes to which the respondents with the same utility function belong. If we obtain the parameters of covariates in the membership function, $\gamma_{s}$, we can interpret why individuals are classified as they are. First, because the marginal utility parameter vector has discrete variation, LCL assures preference heterogeneity. Second, because the ratio of choice probabilities depends not only on the considered alternatives but also on all other alternatives, the IIA property is completely relaxed [45].

The IIA property of a multinomial logit model is expressed by the ratio of choice probabilities related to two alternatives, i and k ([46] (pp. 45–47)). In the context of the maxdiff model, we can arrange the ratio as follows: $P\left(ii^{\prime },i\neq i^{\prime }|M\right)/P\left(kk^{\prime },k\neq k^{\prime }|M\right)$. In the simple maxdiff model, the ratio becomes the form that is independent from unconsidered alternatives: $exp\left(\varphi \left(V\left(i\right)-V\left(i^{\prime }\right)\right)\right)/exp\left(\varphi \left(V\left(k\right)-V\left(k{\;}^{\prime }\right)\right)\right)$. With LCL, the ratio becomes

$$\left(\sum_{s=1}^{S}\pi_{ns}\prod_{t=1}^{T}\frac{exp\left(\varphi \left(V\left(nit|s\right)-V\left(ni^{\prime }\;t|s\right)\right)\right)}{\sum_{\begin{array}{c} j,j^{\prime }\in M\\ j\neq j^{\prime } \end{array}}^{}exp\left(\varphi \left(V\left(njt|s\right)-V\left(nj^{\prime }\;t|s\right)\right)\right)}\right)/\left(\sum_{s=1}^{S}\pi_{ns}\prod_{t=1}^{T}\frac{exp\left(\varphi \left(V\left(nkt|s\right)-V\left(nk^{\prime }\;t|s\right)\right)\right)}{\sum_{\begin{array}{c} j,j^{\prime }\in M\\ j\neq j^{\prime } \end{array}}^{}exp\left(\varphi \left(V\left(njt|s\right)-V\left(nj^{\prime }\;t|s\right)\right)\right)}\right)$$

where it depends on not only the considered alternatives but also all other alternatives. The only exception is when choice probability becomes as follows:

$$\forall s\in S,\sum_{s=1}^{S}\pi_{ns}\prod_{t=1}^{T}\frac{exp\left(\varphi \left(V\left(nit|s\right)-V\left(ni^{\prime }\;t|s\right)\right)\right)}{\sum_{\begin{array}{c} j,j^{\prime }\in M\\ j\neq j^{\prime } \end{array}}^{}exp\left(\varphi \left(V\left(njt|s\right)-V\left(nj^{\prime }\;t|s\right)\right)\right)}=\;\sum_{s=1}^{S}\pi_{ns}\prod_{t=1}^{T}\frac{exp\left(\varphi \left(V\left(nit\right)-V\left(ni^{\prime }\;t\right)\right)\right)}{\sum_{\begin{array}{c} j,j^{\prime }\in M\\ j\neq j^{\prime } \end{array}}^{}exp\left(\varphi \left(V\left(njt\right)-V\left(nj^{\prime }\;t\right)\right)\right)}$$

which represents the degeneration of LCL to the simple maxdiff model in Equation (1).

Third, correlations of parameters may exist because of behavioral phenomena—for example, employers who place high priority on the frequency of payments tend to prefer creating more job opportunities for society—and scale heterogeneity [37]. Especially in the context of scale heterogeneity, cognitive effort-related effects have been suggested, such as learning and fatigue [47,48], choice uncertainty [49], and survey engagement [50]. In the context of household surveys, especially in developing countries, these variabilities can easily occur due to differences in the degree of respondents’ understanding of the scenarios provided in stated preference surveys or their involvement in the survey. Indeed, the covariance matrix of the parameter vector, β, theoretically becomes as follows [37]:

$$Cov\left(\beta \right)=\sum_{s=1}^{S}\pi_{s}\left(\beta_{s}-E\left[\beta \right]\right){\left(\beta_{s}-E\left[\beta \right]\right)}^{\prime }$$

where E[β] denotes the expectation of β globally, or across classes. Therefore, any drawbacks in a multinomial logit model are relaxed by LCL.

Erdem [51] employed a scale-adjusted LCL (SALCL) to take class-specific scale parameters into account; the model was proposed by Vermunt and Magidson [52]. Although class-specific scale parameters can be estimated by SALCL and SALCL appears to be the higher model of the standard LCL, improvements in fit by SALCL are suggested as “simply as a result of increasing distributional flexibility [37] (p. 6)”. Conservatively speaking, SALCL may be a model whose properties are not transparent thus far. Therefore, we decided to employ the standard model of LCL for simplicity.
